# IGF2BP3-induced activation of EIF5B contributes to progression of hepatocellular carcinoma cells

**DOI:** 10.32604/or.2022.026511

**Published:** 2023-01-05

**Authors:** XIAOYIN LI, QIAN WANG, HONGFENG LIANG, SHISHENG CHEN, HAIWEN CHEN, YAOYONG LU, CHANGFU YANG

**Affiliations:** 1 Department of Oncology (Section 3), Gaozhou People’s Hospital, Gaozhou, China; 2 South China Research Center for Acupuncture and Moxibustion, Medical College of Acu-Moxi and Rehabilitation, Guangzhou University of Chinese Medicine, Guangzhou, China; 3 The Second Affiliated Hospital of Guangzhou University of Chinese Medicine (Guangdong Provincial Hospital of Chinese Medicine), Guangzhou, China; 4 Affiliated Nanfang Hospital (Branch Tai He Hospital) of Southern Medical University, Guangzhou, China

**Keywords:** EIF5B, NF-κB, Epithelial-mesenchymal transition, Cancer stem cells, N6-methyladenosine, Hepatocellular carcinoma

## Abstract

In this study, we investigated the functional role of eukaryotic initiation factor 5B (EIF5B) in hepatocellular carcinoma (HCC) and the underlying mechanisms. Bioinformatics analysis demonstrated that the EIF5B transcript and protein levels as well as the EIF5Bcopy number were significantly higher in the HCC tissues compared with the non-cancerous liver tissues. Down-regulation of EIF5B significantly decreased proliferation and invasiveness of the HCC cells. Furthermore, EIF5B knockdown suppressed epithelial-mesenchymal transition (EMT) and the cancer stem cell (CSC) phenotype. Down-regulation of EIF5B also increased the sensitivity of HCC cells to 5-fluorouracil (5-FU). In the HCC cells, activation of the NF-kappa B signaling pathway and IkB phosphorylation was significantly reduced by EIF5B silencing. IGF2BP3 increased the stability of the EIF5B mRNA in an m6A-dependent manner. Our data suggested that EIF5B is a promising prognostic biomarker and therapeutic target in HCC.

## Introduction

According to the World Health Organization (WHO), hepatocellular carcinoma (HCC) is the fifth most common cause of cancer worldwide [1]. The five-year survival rate of HCC is poor because of high rates of recurrence and metastasis [1]. The major risk factors associated with HCC development and progression include chronic hepatitis B or C virus infections, high alcohol consumption, and activation of oncogenes [2]. Therefore, a better understanding of the molecular mechanisms underlying HCC growth and progression is necessary to develop more effective treatment strategies for HCC.

EIF5B (Eukaryotic Translation Initiation Factor 5B) is a conserved eukaryotic translation factor that mediates association of the 40S and 60S ribosomal subunits during eukaryotic translation initiation, and modulates the cell cycle progression by regulating the translation of upstream open reading frames (uORF)-containing mRNAs such as p27 and p21 [3]. In the glioma cells, EIF5B promotes cell survival by enhancing the translation of several IRES-containing mRNAs including those encoding anti-apoptotic proteins such as XIAP and Bcl-xL [4]. EIF5B promotes HCC proliferation and invasion by increasing ASAP1 expression [5]. These data suggest that EIF5B functions as an oncogene that promotes cancer cell growth, survival, and progression. However, the underlying mechanisms by whichEIF5B promotes HCC progression are poorly understood.

Epithelial-mesenchymal transition (EMT) is a dynamic process involved in cancer metastasis wherein cancer cells with epithelial characteristics acquire mesenchymal characteristics such as increased motility, invasion, and survival [6]. Furthermore, cancer cells undergoing EMT phenotypic changes also acquire drug resistance [7]. A subset of cancer cells undergoing EMT acquire characteristics of stem cells and are designated as cancer stem cells (CSCs) [8]. Both EMT and CSC phenotypic changes are associated with tumor recurrence. Therefore, EMT-targeted therapy is necessary to effectively overcome tumor drug resistance and recurrence.

In the present study, we investigated the expression profile and prognostic potential of EIF5B in HCC tissues and cell lines. We also investigated the association between EIF5B expression levels and the EMT and CSC phenotypes in HCC cells using *in vitro* experiments. Furthermore, we investigated the biological functions of EIF5B in the HCC cells by performing *in vitro* EIF5B-silenced HCC cells. Finally, we investigated the mechanisms by which IGF2BP3 regulated EIF5B mRNA stability in the HCC cells.

## Materials and Methods

### Database mining

The EIF5B transcript levels in HCC and normal liver tissues were analyzed from datasets in the HCCDB [9], Oncomine, UALCAN [10] and GEPIA [11] databases.

### Cell culture and HCC tissue samples

HepG2 cells were purchased from the Cell Bank of Type Culture Collection (Chinese Academy of Sciences, Shanghai, China) and used for *in vitro* experiments. We also obtained 16 pairs of HCC and adjacent normal liver tissue samples from the Gaozhou People’s Hospital, Maoming, China. The details of the patients enrolled in the study were listed in Suppl. Table 2.

### Cell transfection

We seeded 2 × 10^5^ HepG2 cells per well in six-well plates and transfected them with small interfering RNAs targeting EIF5B (siEIF5B-1: 5′-TCCTGATATTTGGGCTTAG-3′; siEIF5B-2: 5′CCTGATATTTGGGCTTAGT-3′) or scrambled nonsense siRNA (si-NC) using lipofectamine 2000 (Invitrogen, catalog number: 11668, USA) according to the manufacturer’s instructions. 48 h after transfection, cells were subjected to the following experiments.

### Cell transduction

We cloned the short hairpin RNA (shRNA) against EIF5B (5′-GGGCTTAGTGCTTCTAAATTG-3′) into the lentiviral vector (pENTR™/H1/TO, Invitrogen, USA) and packaged the recombinant lentiviral vector according to the manufacturer’s protocol. HCC cells were then infected with the lentiviral particles containing shEIF5B or sh-NC. 72 h after transduction, we harvested the cells and used them for subsequent experiments.

### MTT assay

The viability of control and EIF5B-silenced HCC cells (2 × 10^5^ per well) was determined by the 3-(4,5-dimethylthiazol-2-yl)-2,5-diphenyl tetrazolium bromide (MTT) (Sigma, catalog number: M5655, USA) assay. Briefly, HCC cells were seeded in a 96-well plate. At specific time points (0, 24, 48, 72, 96, and 10 h), 10 µl of MTT solution was added per well and the cells were further incubated for 4 h at 37°C.Then, the absorbance was measured at OD = 590 nm. Cell viability was measured with appropriate controls.

### Colony formation assay

The control (si-ctrl) and EIF-5B-silenced (si-EIF5B) cells were seeded into at a density of 200 cells per well in six-centimeter petriplates and cultured for 14 days. Then, the colonies were washed with phosphate-buffered saline (PBS), fixed with methanol and stained with 0.1% crystal violet solution. Then, the total number of colonies was estimated under a light microscope.

### Transwell assay

The control (si-ctrl) or EIF5B-knockdown (si-EIF5B) cells in serum-free medium (seeding density: 1 × 10^5^) were seeded in the upper chamber of the Transwell (8 μm, Coring, Cambridge, MA, USA). The lower chamber was filled with medium containing 10% FBS as a chemo attractant. After 20 h, the cells in the top chamber were removed. The cells in the bottom chamber (migrated) were stained with 0.1% crystal violet staining solution and the total number of invasive cells were counted under a light microscope. When counting the transwell cell, we used the software called CELLCOUNTER as previously described. The algorithm used was *h* × [*L*/2*r* + *a*] × [*W*/2*r* + *a*], in which L and W are the length and width of the minimum bounding rectangle, h is the number of layers that the cells stack, and a is a parameter to account for cells at the boundary of the rectangle.

### Flow cytometry

Flow cytometry was used to analyze the cell cycle and ALDH1 activity in the control and EIF5B-silenced HCC cells. For the cell cycle analysis, the control and EIF5B-silenced HCC cells were fixed overnight with 70% ethanol at 4°C. Then, the fixed cells were washed with phosphate buffered saline (PBS), stained with propidium iodide, and analyzed by flow cytometry. The ALDH1 activity in the control and EIF5B-silenced HCC cells was analyzed by flow cytometry as previously described [12]. Briefly, cells were prepared as a single cell suspension for staining. The ALDH activity was assayed using the ALDEFLUOR Kit and following the manufacturer’s instructions (Stemcell, Catalog number: #01700, Canada). Diethylaminobenzaldehyde (DEAB) was added to each sample as a negative control. After staining, cells were washed twice with PBS, followed by FACS analysis using a FACSAria Flow cytometer.

### Tumor sphere formation

The CSC phenotype of the control and EIF5B-silenced HCC cells was analyzed by the tumor sphere formation assay as previously described [13]. Briefly, single cells were washed with PBS. Subsequently, single cells were plated in Ultra Low Attachment plates (Corning) in serum-free DMEM-F12 supplemented with 10 ng/mL bFGF, 10 ng/mL EGF, and B27 (all from Invitrogen). In such conditions, cells formed tumor sphere.

### Estimation of NF-κB activity

The cells were co-transfected with a NF-κB reporter plasmid and an internal control vector. Then, the cells were stimulated with TNF-α (catalog number: P6231, Beyotime Biotechnology, China) for 24 h. The firefly and Renilla luciferase activities were measured for all the groups in a luminometer. The relative firefly luciferase activity (NF-κB activity) was determined by normalization to the Renilla luciferase activity.

### Western blot assay

Total cell protein lysates were extracted from the control and EIF5B-silenced HCC cells with the RIPA buffer (catalog number: P0013C, Beyotime Biotechnology, China). Protein concentration was analyzed using the BCA assay. Equal amount of protein samples were separated on a 10% SDS–PAGE. The separated proteins were transferred onto a PVDF membrane. The membrane was then blocked with 5% non-fat milk. Then, the blots were incubated overnight with primary antibodies at 4°C. Subsequently, after washing with 1X TBST buffer, the blots were incubated with the secondary antibodies for 1 h at 37°C. Then, the blots were developed by enhanced chemiluminescence using an ECL kit and the protein bands were analyzed. The concentration and the Lot numbers of the primary antibodies used were list as following: phospho-IKB alpha (Absin Bioscience, abs155093), GAPDH (abcam, ab8245), EIF5B (abcam, ab89016), CDK4 (abcam, ab108357), CDK6 (abcam, ab124821), MMP2 (abcam, ab92536), MMP9 (abcam, ab76003), E-cadherin (abcam, ab40772), N-cadherin (abcam, ab76011), Vimentin (abcam, ab8978), phospho-IKK (abcam, ab194528), SOX2 (abcam, ab92494) and NOTCH2 (abcam, ab245699). Concentration: 1:500.

### Immunofluorescence staining

HCC cells were seeded to the culture slides. 24 h later, cells were rinsed with PBS, followed by fixed with paraformaldehyde. Then the cells were blockade in 5% BSA. After incubation with the primary antibody against E-cadherin, cells were washed in PBS, followed by incubated with the second antibody. The nuclear was detected by DAPI dye.

### RNA stability assays

The control and EIF5B cells were treated with actinomycin D at 0, 3, and 6 h. Subsequently, RNA samples were extracted at all the time points and analyzed by the RT-qPCR assay. The expression levels of EIF5B mRNA and other mRNAs were normalized to β-Actin mRNA levels.

### RNA immunoprecipitation (RIP) assays

The RNA immunoprecipitation assay was performed using the Magna RIP RNA-Binding Protein Immunoprecipitation Kit (catalog number: 17-704, Millipore, USA) according to the manufacturer’s instructions. In brief, magnetic beads were coated with immunoglobulin G or IGF2BP3. Then, the coated beads were incubated overnight at 4°C with the cell lysates. The complexes were then washed and subjected to proteinase K digestion. Then, the transcripts were analyzed by the qPCR assay to determine the interaction between IGF2BP3 and EIF5B transcripts.

### MeRIP qPCR

Dyna beads™ mRNA Purification Kit (catalog number: 61006, Invitrogen, USA) was used to purify the poly(A) mRNA from the total cellular RNA. Then, the Pierce™ Protein A/G magnetic beads were incubated withanti-m6A antibody or IgG for conjugation according to the manufacturer’s instructions. The antibody-conjugated beads were then incubated with the poly(A) mRNA samples. Subsequently, the methylated mRNAs were precipitated with glycogen after proteinase K digestion and subjected to quantitative PCR to quantify the enrichment of specific mRNAs.

### Statistical analysis

SPSS software version 13.0 (SPSS Inc., Chicago, Illinois, USA) and Graph Pad Prism software version 5.0 (GraphPad software Inc, San Diego, California, USA) were used for statistical analysis. The data are represented as means ± S.E.M. The differences between groups were analyzed by one-way ANOVA or two-tailed Student’s *t*-test. *p* < 0.05 was considered statistically significant.

## Results

### EIF5B transcripts, copy number, and protein levels are elevated in HCC tissues

**Figure 1:**
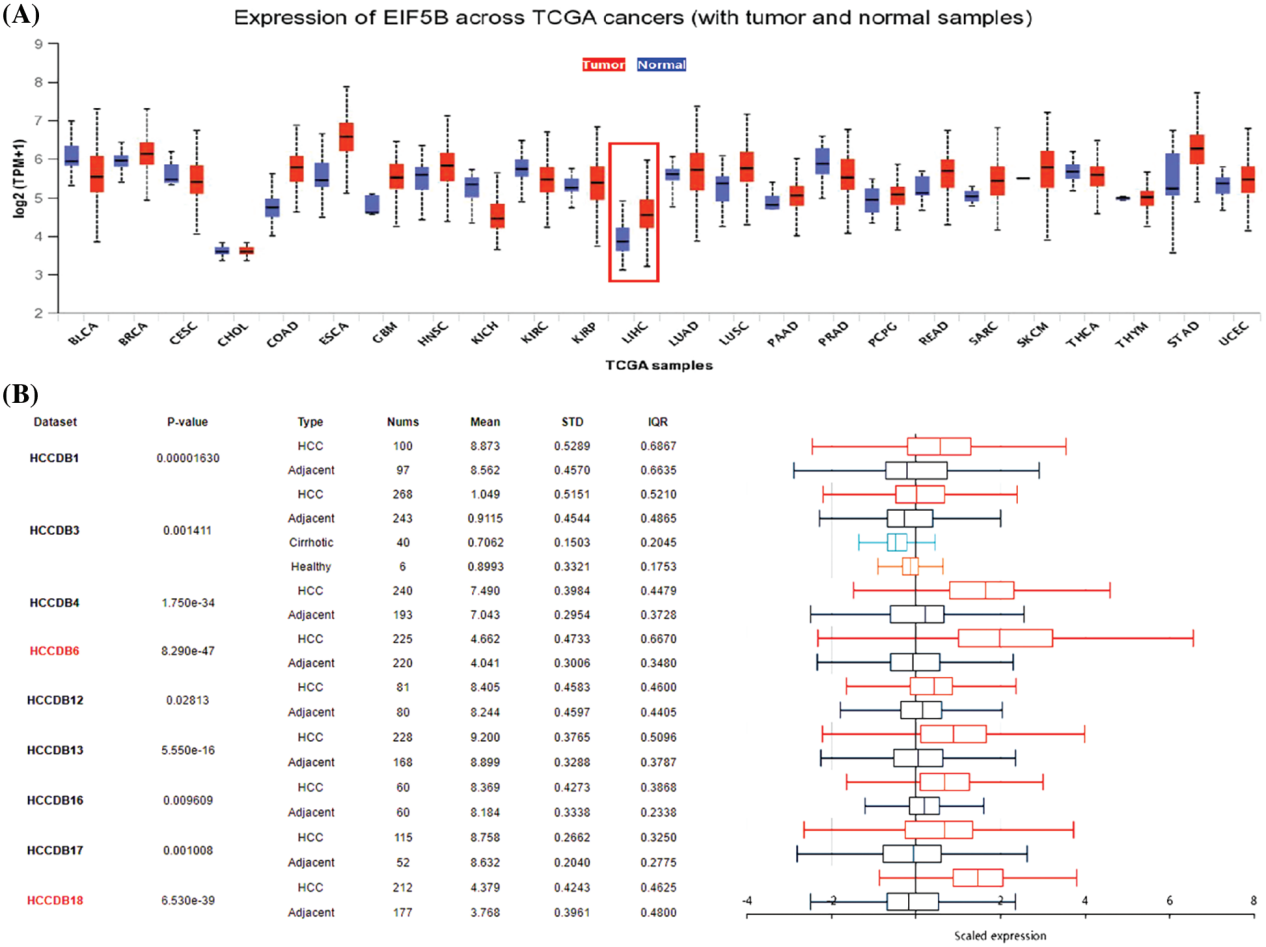
The expression levels of EIF5B transcripts and mRNA in HCC tissues. (A) The transcription level of EIF5B in HCC tissues and non-cancer liver tissues, as demonstrated by UALCAN database. (B) The HCCDB database revealed that the mRNA expression level of EIF5B was significantly elevated in HCC tissues.

We first compared the expression levels of EIF5B in HCC and normal liver tissues by analyzing the RNA-seq data from the HCCDB database (GEO datasets), UALCAN database (TCGA datasets) and the Oncomine database. UALCAN database analysis demonstrated that the EIF5B transcript levels were significantly higher in the tumor tissues from multiple cancers including HCC compared to the corresponding non-cancer tissues (Fig. 1A, *p* < 0.05). HCCDB database analysis demonstrated that the EIF5B mRNA levels were significantly higher in the HCC tissues compared to the adjacent non-cancerous liver tissues (Fig. 1B, *p* < 0.05).

**Figure 2:**
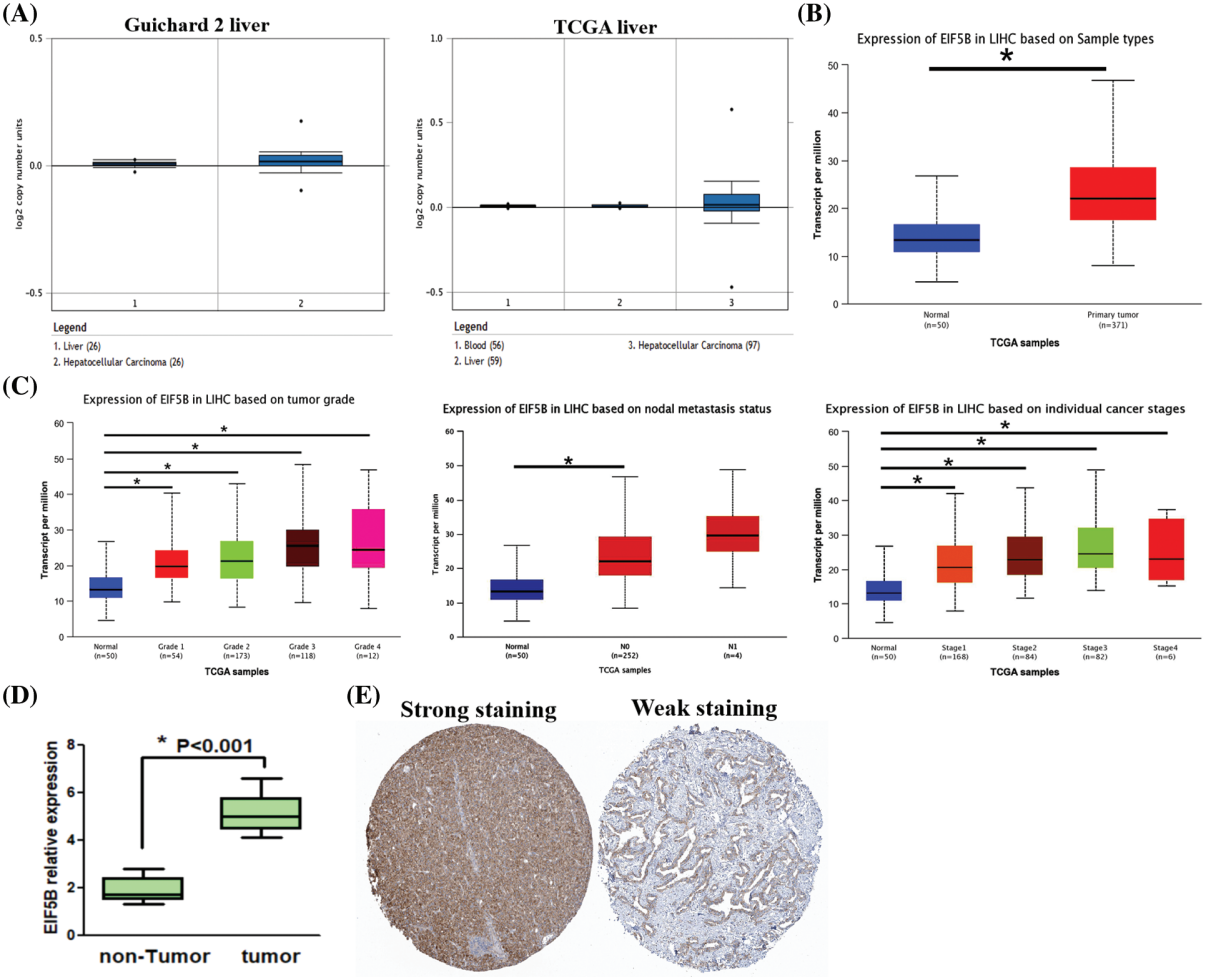
The expression levels of EIF5B copy number and protein in HCC tissues. (A) The Oncomine database revealed that copy numbers of EIF5B were significantly increased in HCC tissues when compared with non-HCC tissues. (B) The transcription levels of EIF5B were significantly increased in HCC tissues when compared with non-HCC tissues, indicated by UALCAN database. (C) The transcription levels of EIF5B and multiple clinic-pathological features were indicated by UALCAN database. (D) RT-PCR assay was used to analyze mRNA expression level in HCC tissues. x axis: tissue samples; y axis: EIF5B relative expression (n = 16). (E) The immunohistochemistry assay demonstrated that EIF5B was also located to the cytosol in HCC tissues (data from Human Protein Atlas database). All the experiment replicated 3 times. * represented *p* < 0.05. All the error bars stand for the mean ± standard deviation (SD).

Oncomine database analysis demonstrated that the copy numbers of EIF5B were significantly higher in the HCC tissues compared with the non-HCC liver tissues (Fig. 2A, *p* < 0.05). The EIF5B transcript levels in the HCC tissues from the UALCAN database are shown in Fig. 2B. The transcript levels of EIF5B in the HCC tissues based on multiple clinicopathological features are shown in Fig. 2C. With the use of RT-PCR assay, we found that EIF5B expression level was elevated in HCC tissues (Fig. 2D). We next analyzed the EIF5B protein levels in HCC tissues using the Human Protein Atlas database. Immunohistochemical analysis of HCC tissues demonstrated that EIF5B protein was localized in the cytosol (Fig. 2E). Taken together, these data demonstrated that EIF5B was over-expressed in HCC tissues compared to the non-cancerous liver tissues.

### Overexpression of EIF5B is associated with worse DFS and OS in HCC patients

**Figure 3:**
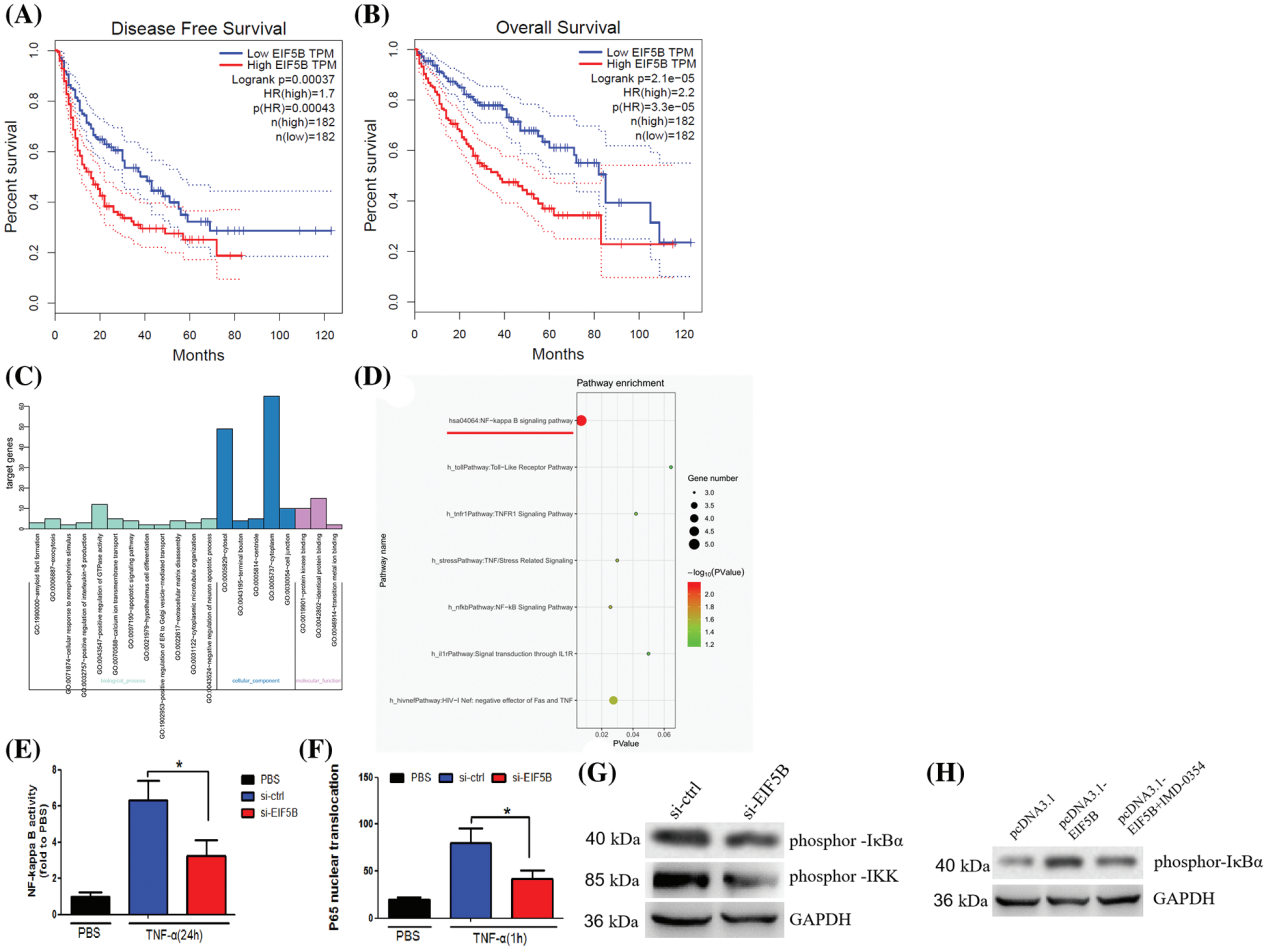
Overexpression of EIF5B predicts poor prognosis of HCC and activate NF-κB pathway activity. (A) Survival curves of HCC patients DFS rate with high- and low-EIF5B expression levels. (B) Survival curves of HCC patients OS rate with high- and low-EIF5B expression levels. (C) The genetic ontology analysis revealed the altered pathways that may be involved in EIF5B’s biological behavior. (D) KEGG pathway analysis revealed that the altered genes were involved in NF-κB pathway. (E) EIF5B down-regulation decreased NF-κB pathway transcriptional activity when stimulated by TNF-α. x axis: treatment, y axis: NF-κB pathway transcriptional activity (N = 3, *p < 0.05, Student’s *t*-test). (F) Silencing of endogenous EIF5B resulted in retention of most of the P65 in the cytoplasm upon TNF-α stimulation. x axis: treatment, y axis: P65 activity (N = 3, *p < 0.05, Student’s *t*-test). (G) Down-regulation of EIF5B decreased phosphorylation at Ser-32 ofIκBα and phosphorylation of IKK. (H) Overexpression of EIF5B increased phosphorylation at Ser-32 of IκBα. All the error bars stand for the mean ± standard deviation (SD).

HCC samples in the GEPIA database were analyzed to determine the relationship between expression levels of EIF5B and the disease-free survival (DFS) and overall survival (OS) rates in HCC patients. The HCC patients were classified into high and low expression groups based on the median value of EIF5B expression. HCC patients with highEIF5B expression showed poorer DFS and OS rates than those with low EIF5B expression (Figs. 3A and 3B). This suggested that EIF5B was a potential prognostic biomarker for HCC patients.

### EIF5B silencing inhibits NF-κB signaling pathway in the HCC cells

Next, we analyzed the underlying mechanisms by which EIF5B augments HCC growth and progression. HepG2 cells were transfected with two siRNAs targeting EIF5B. The siEIF5B-1 oligo showed better knockdown efficiency than the siEIF5B-2 oligo. Therefore, siEIF5B-1 was chosen for further analysis. The expression profiles of control and EIF5B-silenced HepG2 cells were analyzed and the differentially expressed genes (up-regulated or down-regulated with a fold change ≥2-fold) were identified as shown in Suppl. Table 1. Gene ontology analysis was performed to identify the enriched GO terms associated with EIF5B expression in HCC cells (Fig. 3C). KEGG pathway analysis demonstrated that EIF5B expression changes in HCC cells were associated with the NF-κB pathway (Fig. 3D).

EIF5B silencing decreased the transcriptional activity related with the NF-κB signaling pathway when the control and EIF5G-silenced HepG2 cells were stimulated by TNF-α (Fig. 3E). TNF-α-stimulated EIF5B-silenced HepG2 cells stimulated showed significantly high levels of P65 protein in the cytoplasm compared to the corresponding controls (Fig. 3F). The IKK (inhibitor of NF-κB kinase) complex has an essential role in the activation of the family of NF-κB transcription factors. IκBα tightly regulates the transcriptional activity of NF-κB by retaining it in the cytoplasm in an inactive form. We then analyzed the underlying mechanisms by which EIF5B regulated activation of NF-κB by evaluating the expression and phosphorylation levels of IκBα and IKK in the control and EIF5B-silenced HepG2 cells. Western blotting showed that EIF5B down-regulation decreased phosphor IκBα (Ser 32) levels and increased the phosphorylation levels of IKK (Fig. 3G). In addition, overexpression of EIF5B increased phosphorylation at Ser-32 of IκBα (Fig. 3H). This demonstrated that EIF5B silencing decreased NF-κB activation in the HCC cells by inhibiting IκBα phosphorylation.

### EIF5B promotes growth and cell cycle progression of HCC cells via CDK4 and CDK6

**Figure 4:**
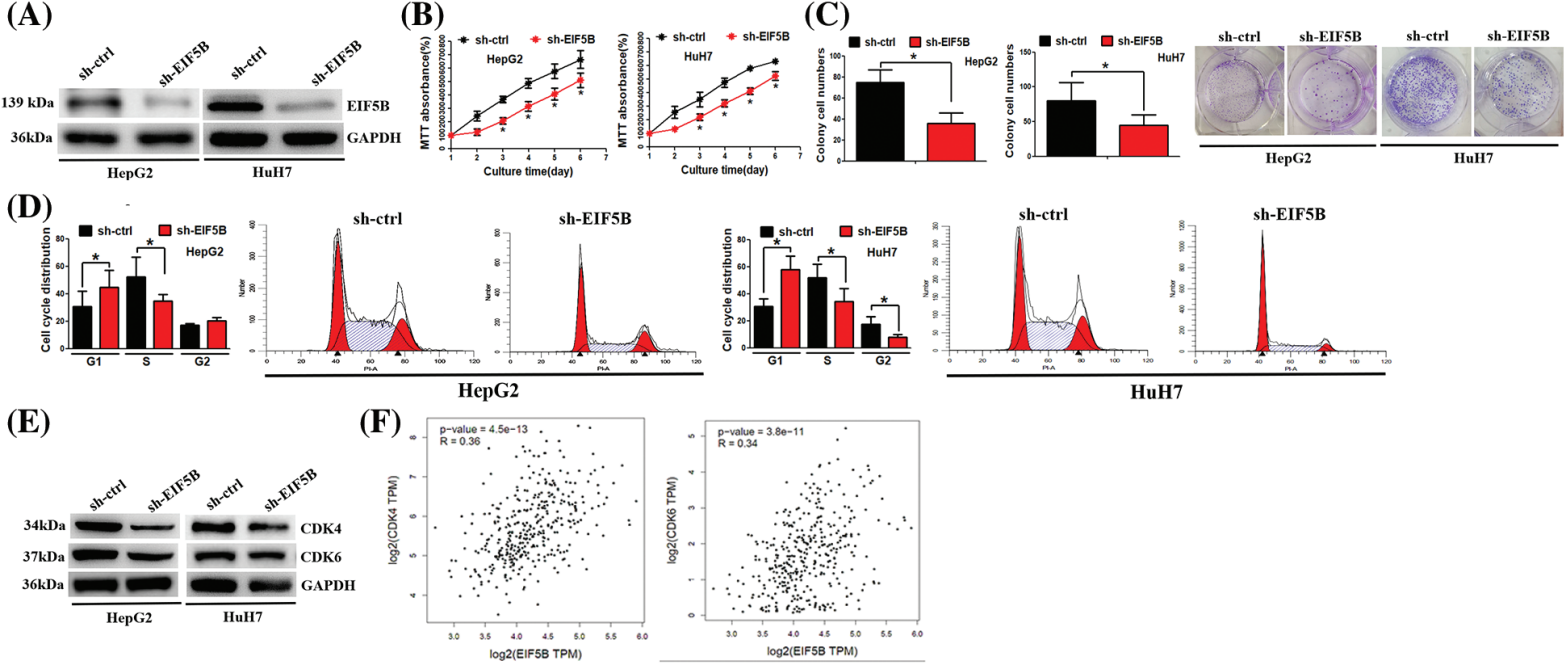
Down-regulation of EIF5B decreased HCC cell growth ability *in vitro*. (A) The knock-down efficiency of EIF5B was examined by western blot assay. (B) The MTT assay revealed that down-regulation of EIF5B decreased HCC cell proliferation. x axis: culture time, y axis: MTT absorbance (N = 3, *p < 0.05, Student’s *t*-test). (C) The colony formation assay revealed that down-regulation of EIF5B decreased HCC cell colony formation ability. x axis: treatment group, y axis: colony cell numbers (N = 3, *p < 0.05, Student’s *t*-test). (D) Down-regulation of EIF5B induced cell cycle arrest at G1 phase. x axis: cell cycle distribution, y axis: cell cycle distribution rate (N = 3, *p < 0.05, one-way analysis of variance (ANOVA)). (E) The expression levels of CDK4 and CDK6 were decreased in EIF5B down-regulation cells. (F) The expression level of EIF5B was positively associated with expression levels of CDK4 and CDK6 in HCC, as revealed by the GEPIA database. All the error bars stand for the mean ± standard deviation (SD).

Next, we analyzed whether down-regulation of EIF5B reduced HCC cell growth and cell cycle progression. We generated stable EIF5B-silencedHCC cells using lentiviral vectors containing EIF-5B-specific shRNA. The knockdown efficiency of the EIF-5B-specific shRNA was confirmed by western blotting analysis (Fig. 4A).

MTT and colony formation assay results showed that down-regulation of EIF5B significantly reduced the proliferation (Fig. 4B) and colony formation (Fig. 4C) of HCC cells. Furthermore, cell cycle analysis showed that down-regulation of EIF5B induced G1cell cycle arrest in the HCC cells (Fig. 4D). The expression levels of cell cycle promoters such as CDK4 and CDK6 were significantly reduced in the EIF5B knockdown HCC cells compared to the corresponding controls (Fig. 4E). EIF5B expression levels showed positive correlation with CDK4 and CDK6 expression levels in the HCC patients from the GEPIA database (Fig. 4F). These results showed that EIF5B promoted cell cycle progression and growth of HCC cells via CDK4 and CDK6.

### EIF5B promotes HCC progression by inducing EMT

**Figure 5:**
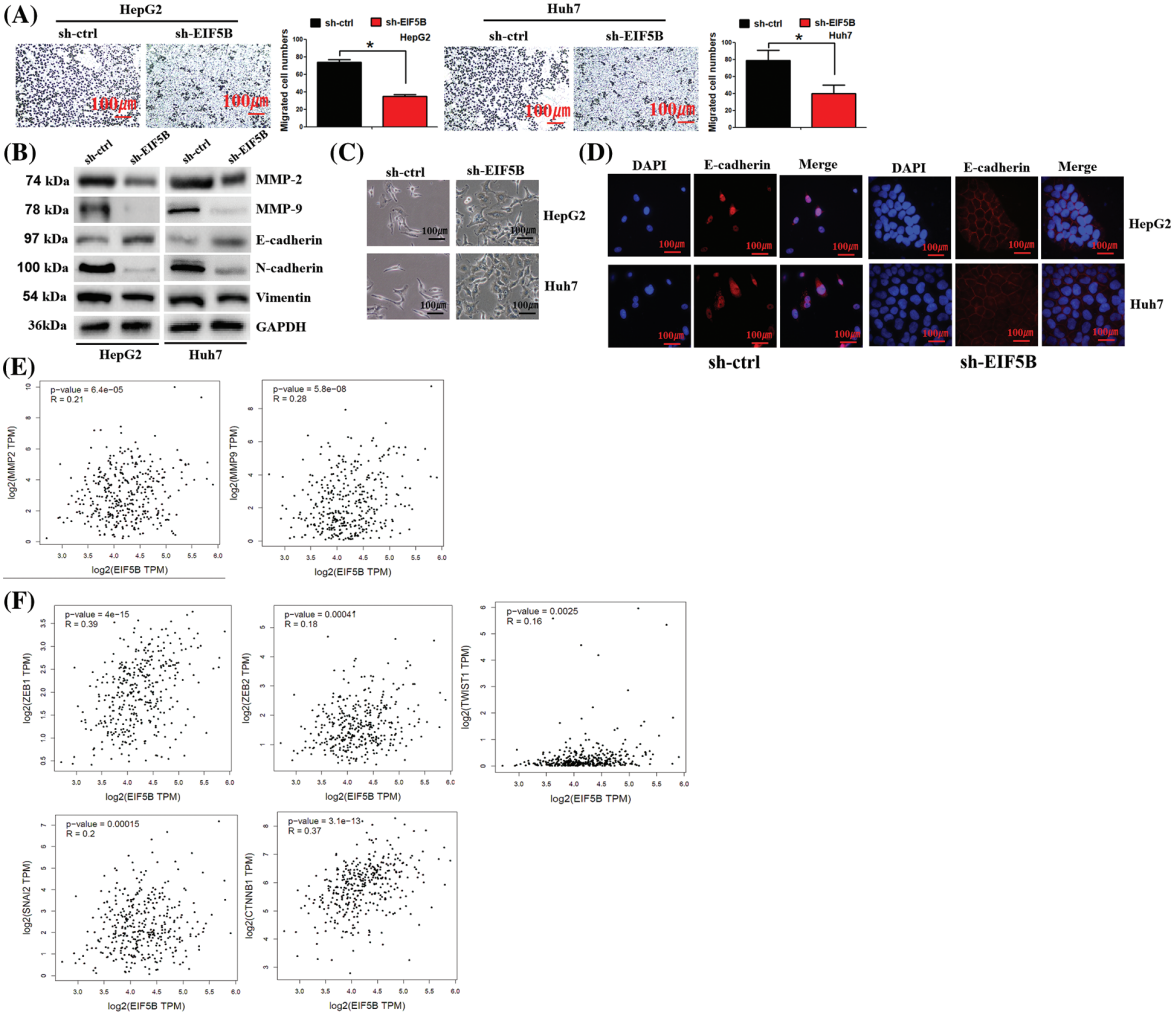
Down-regulation of EIF5B decreased HCC cell migration ability *in vitro*. (A) The transwell assay found that down-regulation of EIF5B inhibited HCC cell migration. x axis: treatment group, y axis: migrated cell numbers (N = 3, *p < 0.05, Student’s *t*-test). (B) Down-regulation of EIF5B decreased the expression levels of MMP-2, MMP-9, N-cadherin and Vimentin, but elevated expression level of E-cadherin, revealed by the western blot assay. (C) The phenotypic changes in sh-ctrl and sh-EIF5B cells were demonstrated. (D) IF staining of E-cadherin location in sh-ctrl and sh-EIF5B cells were demonstrated. DAPI was used to stain DNA (blue), anti-rabbit secondary conjugated with Alexa594 was used to stain E-cadherin (red). (E) EIF5B expression level was positively associated MMP-2 and MMP-9 expression levels. (F) EIF5B expression level was positived associated the EMT inducer (e.g., ZEB1, ZEB2, snail2, twist1 and CTNNB1). All the error bars stand for the mean ± standard deviation (SD).

We then analyzed the effects of EIF5B downregulation on the migration of HCC cells. Transwell assay results showed that down-regulation of EIF5B inhibited *in vitro* migration of HCC cells (Fig. 5A). Matrix metalloproteinases (MMPs) are associated with the migration and invasiveness of cancer cells [14]. Therefore, we analyzed the expression levels of matrix metalloproteinases, MMP-2 and MMP-9, in the control and EIF5B-silencedHCC cells. Our results showed that down-regulation of EIF5B decreased the expression levels of MMP-2 and MMP-9 in the HCC cells (Fig. 5B).

We then examined the effects of EIF5B downregulation on the EMT phenotype of HCC cells. EIF5B down-regulation increased expression levels of the mesenchymal cell marker, E-cadherin, and decreased the expression levels of the epithelial cell markers, N-cadherin and Vimentin (Fig. 5B). This demonstrated that EIF5B downregulation inhibited EMT. Interestingly, we observed phenotypic changes in cells when EIF5B was down-regulated. In sh-ctrl group, cells presented spindle-shaped morphology, which is feature of EMT. However, in sh-EIF5B group, cells presented cuboidal-shaped morphology, which suggested the reverse of EMT (Fig. 5C). EMT process was characterized by decreased membranous E-cadherin and translocation of E-cadherin to the nucleus. We further examined E-cadherin location by immunofluorescence assay. As expected, E-cadherin was mainly located in nucleus in sh-ctrl cells. However, in sh-EIF5B cells that EMT was inversed, E-cadherin located mainly in membrane (Fig. 5D).

EIF5B expression levels showed positive correlation with MMP-2 and MMP-9 expression levels in the HCC patients from the GEPIA database (Fig. 5E). Furthermore, EIF5B expression levels were associated with the expression levels of EMT inducers such as ZEB1, ZEB2, snail2, twist1 and CTNNB1 (Fig. 5F).

Taken together, these data demonstrated that EIF5B promoted HCC migration and invasion by inducing the EMT phenotype and upregulating the expression of MMPs. These effects were reversed by the downregulation of EIF5B.

### EIF5B promotes cancer stem cell phenotype in HCC

**Figure 6:**
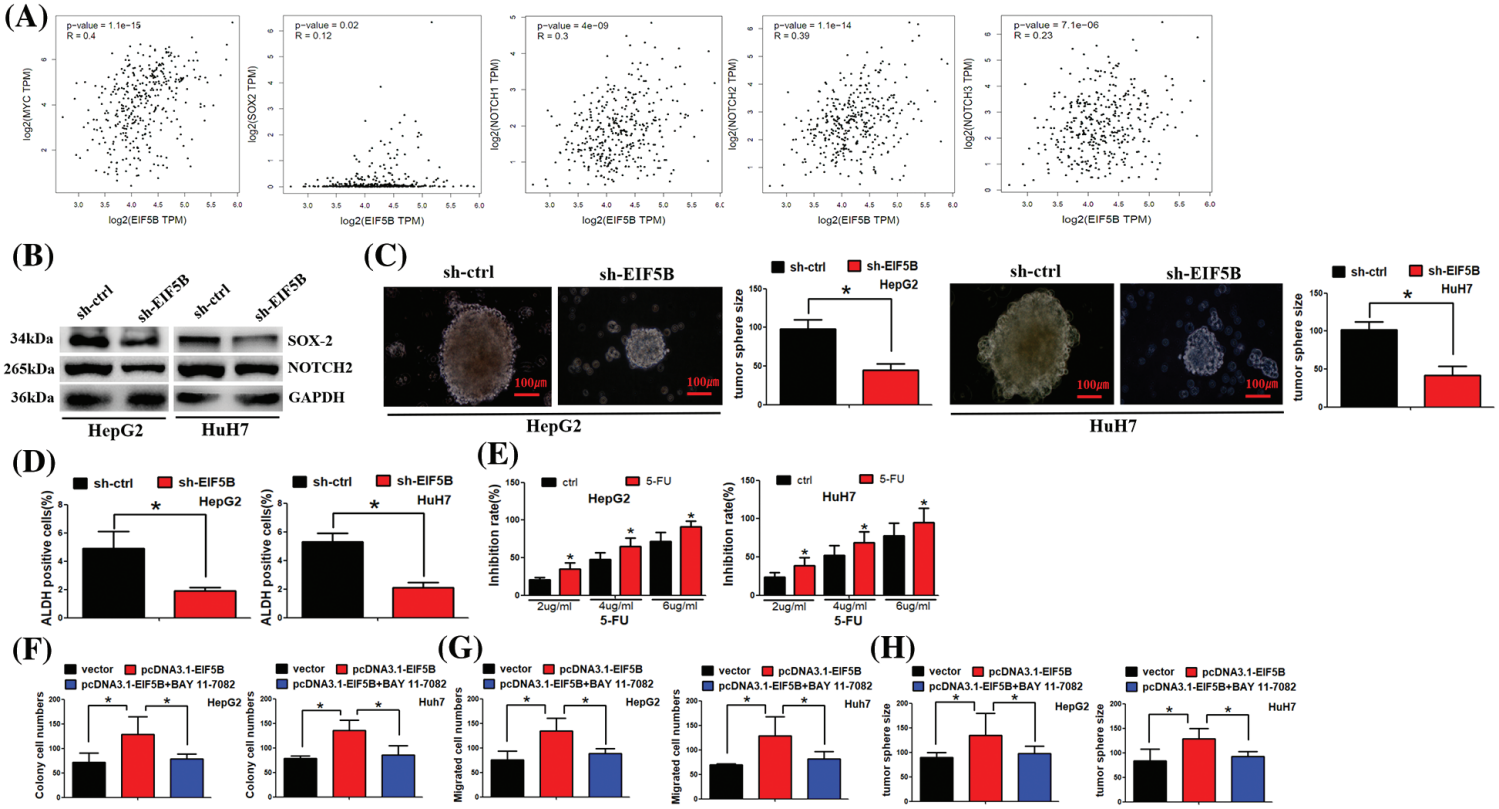
Down-regulation of EIF5B decreased cancer stem cells (CSCs) properties in HCC. (A) EIF5B expression level was positived associated the CSCs markers (e.g., myc, SOX2, NOTCH1, NOTCH2 and NOTCH3) in HCC tissues. (B) Western blot assay was carried out to examine protein expression levels. (C) N = 3. The tumor sphere formation assay revealed that EIF5B down-regulation impaired the tumor sphere formation ability of HCC cells. x axis: treatment group, y axis: tumor size (N = 3, *p < 0.05, Student’s *t*-test). (D) The ALDH1 activity indicated that ALDH1 activity was decreased when EIF5B was knocked down. x axis: treatment group, y axis: ALDH positive cells (N = 3, *p < 0.05, Student’s *t*-test). (E) Cell viability assay showed that knockdown of EIF5B increased 5-FU sensitivity in HCC cells. x axis: drug concentration, y axis: inhibition rate (N = 3, *p < 0.05, one-way analysis of variance (ANOVA)). (F) Colony formation assay was performed. x axis: treatment group, y axis: colony cell numbers (N = 3, *p < 0.05, one-way analysis of variance (ANOVA)). (G) Transwell assay was performed to analyze cell migration ability. x axis: treatment group, y axis: migrated cell numbers (N = 3, *p < 0.05, one-way analysis of variance (ANOVA)). (H) Tumor sphere was cultured to analyze cancer stem cell property. x axis: treatment group, y axis: tumor sphere size (N = 3, *p < 0.05, one-way analysis of variance (ANOVA)). All the error bars stand for the mean ± standard deviation (SD).

GEPIA database analysis demonstrated that EIF5B expression levels were positively associated with the expression levels of the CSC markers such as c-Myc, SOX2, NOTCH1, NOTCH2 and NOTCH3 in the HCC tissues (Fig. 6A). Western blot analysis confirmed that down-regulation of EIF5B significantly reduced the expression levels of CSC marker proteins such as c-Myc, SOX2, NOTCH1, NOTCH2 and NOTCH3 in the HCC cells (Fig. 6B). We next analyzed the effects of EIF5B downregulation on the CSC phenotype of HCC cells using the tumor sphere formation assay. EIF5B down-regulation significantly impaired tumor sphere formation ability of the HCC cells (Fig. 6C). ADH1 is a functional marker of CSCs. EIF5B downregulation significantly reduced ALDH1 activity in the HCC cells (Fig. 6D). Clinically, 5-fluorouracil (5-Fu) is a chemotherapeutic drug that is commonly used for the treatment of HCC patients. However, HCC patients usually develop resistance to 5-Fu and this phenomenon affects 5-Fu efficiency in HCC treatment. Thus, we asked whether EIF5B downregulation could sensitize HCC cells to 5-Fu. Furthermore, cell viability assay showed that EIF5B knockdown increased 5-FU sensitivity of the HCC cells (Fig. 6E). Taken together, these data demonstrated that down-regulation of EIF5B decreased the CSC phenotype of HCC cells and enhanced their 5-FU sensitivity.

We further asked whether EIF5B exerted its function via NF-κB pathway. As expected, overexpression of EIF5B increased colony formation, cell migration and tumor sphere formation ability. However, co-treatment of EIF5B and BAY 11-7082 (which antagonizes NF-κB activation) dismissed overexpression of EIF5B effect on HCC cells (Figs. 6F–6H).

### IGF2BP3 enhances EIF5B mRNA stability via an m6A-dependent manner in HCC

**Figure 7:**
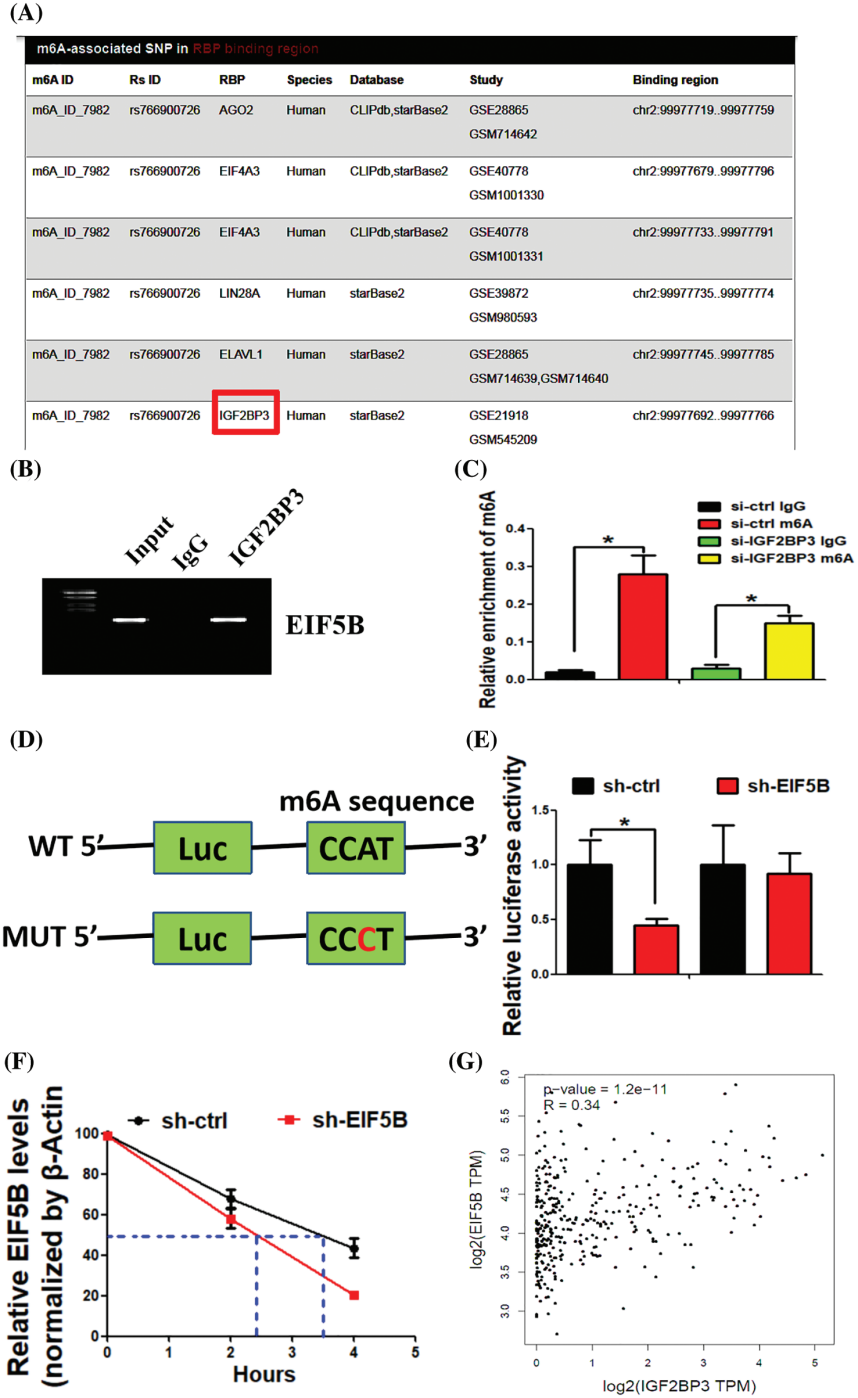
IGF2BP3 enhances EIF5B mRNA stability via an m6A-dependent manner. (A) The biding of IGF2BP3 with EIF5B was indicated. (B) The direct binding between the IGF2BP3 protein and EIF5B mRNA was demonstrated by RIP assay. (C) MeRIP-qPCR assay revealed that IGF2BP3 inhibition decreased m6A levels of EIF5B. x axis: treatment group, y axis: m6A enrichment (N = 3, *p < 0.05, one-way analysis of variance (ANOVA)). (D) The luciferase reporters contained WT (wild type) or MUT (mutant type) m6A consensus sequences of EIF5B were indicated. (E) Knockdown of IGF2BP3 decreased the transcriptional level of wild-type EIF5B. x axis: treatment group, y axis: luciferase activity (N = 3, *p < 0.05, one-way analysis of variance (ANOVA)). (F) The decay rate of mRNA and PCR assay of EIF5B at the indicated times afteractinomycin D (5 μg/ml) treatment in HCC cells after IGF2BP3 inhibition. x axis: hours, y axis: relative EIF5B expression (N = 3, *p < 0.05, one-way analysis of variance (ANOVA)). (G) Correlation between IGF2BP3 and EIF5B expression in TCGAdatabase of HCC. All the error bars stand for the mean ± standard deviation (SD).

N^6^-methyl adenosine (m6A) modification is associated with mRNA stability and is dysregulated in HCC. Therefore, we analyzed if m6A modification contributed to elevated expression levels of EIF5B in the HCC cells. The m6AVar database (http://m6avar.renlab.org/) analysis identified potential m6A modification sites in the EIF5B mRNA. IGF2BP3 is a RNA binding protein (RBP) that interacts with EIF5B [15]. Fig. 7A shows the binding of IGF2BP3 with EIF5B mRNA. RIP assays validated the direct interaction between IGF2BP3 and EIF5B mRNA in the HCC cells (Fig. 7B). MeRIP-qPCR assay results demonstrated that enrichment of EIF5B mRNA by the m6A-specific antibody was significantly decreased in the lysates from the si-IGF2BP3-tranfected HCC cells (Fig. 7C). Luciferase reporter assay was performed with the wild-type or mutant EIF5B to determine the effects of m6A modification on EIF5B expression. The mutant EIF5B was constructed by replacing the adenosine bases by cytosine in the m6A consensus sequences (RRACH) of EIF5B mRNA. Luciferase reporter activity was significantly reduced in the HCC cells with mutant EIF5B but the luciferase activity was maximal in the HCC cells with wild-type EIF5B (Fig. 7D). Furthermore, knockdown of IGF2BP3 in the HCC cells decreased the transcript levels of wild type EIF5B but did not affect the transcript levels of the mutant EIF5B (Fig. 7E). These results confirmed that IGF2BP3 mediated the m6A modification of EIF5B.

We subsequently analyzed the EIF5B mRNA decay rates in the si-IGF2BP3- and si-ctrl-transfected HCC cells. IGF2BP3-silenced HCC cells showed significantly reduced expression of the EIF5B mRNA; moreover, the half-life of the EIF5B mRNA was significantly shortened in the IGF2BP3-silenced HCC cells compared to the controls (Fig. 7F). In the HCC samples from the TCGA database, IGF2BP3 levels showed positive correlation withEIF5B levels (Fig. 7G). These data demonstrated a positive regulatory mechanism between IGF2BP3 and EIF5B in HCC.

## Discussion

EIF5B is the eukaryotic or tholog of IF-2, the bacterial translation initiation factor-2. It plays a key role in the stabilization of the association between initiator methionyl tRNA (tRNA-Meti) and the ribosome. Several studies have reported that EIF5B functions as an oncogene and promotes cancer progression. In glioblastoma cell lines, EIF5B suppressed apoptosis by promoting the translation of pro-survival and anti-apoptotic proteins [5]. EIF5B promotes HCC cell proliferation, migration and invasiveness by enhancing ASAP1 expression [5].

We analyzed the EIF5B transcript and protein levels, and *EIF5B* copy number in HCC and non-cancerous liver tissues from multiple databases. The transcript levels, copy number, and protein levels of EIF5B were significantly increased in the HCC tissues compared with the non-cancer liver tissues. GEPIA database analysis demonstrated that EIF5B overexpression was associated with worse DFS and OS in the HCC patients. This finding was similar to a previous report [5].

The underlying mechanisms by which EIF5B regulates HCC cell growth and progression is poorly understood. Therefore, we used specific siRNAs targeting EIF5B to downregulate the expression levels of EIF5B in the HCC cells. Then, we performed RNA-sequencing analysis of the control and EIF5B-silenced HCC cells to determine the differentially expressed genes (DEGs) that are regulated by EIF5B. KEGG pathway analysis of the DEGs demonstrated that EIF5B knockdown altered several genes in the NF-kappa B signaling pathway. NF-κB is a master transcriptional regulator of cell survival and cancer cell progression, including HCC cells [16]. In HCC, irrespective of the etiology, activation of NF-κB is an early event that is linked with liver cell transformation during hepatocarcinogenesis [17]. Our data demonstrated that EIF5B down-regulation decreased NF-κB activation by inhibiting IκBα phosphorylation.

MTT and colony formation assays showed that down-regulation of EIF5B inhibited HCC cell growth. Furthermore, EIF5B down-regulation induced cell cycle arrest at the G1 phase by decreasing the expression levels of cell cycle promoters such as E2F1, CDK4, and CDK6. In the analysis from GEPIA database, the correlation between EIF5B and CDK4 and CDK6 expression was not as strong as expected, though the correlation was positive. However, it is worthy to note that the regulatory relationship in mechanism does not necessarily represent the degree of correlation. Transwell assay results showed that EIF5B silencing decreased the migration of HCC cells by suppressing EMT and decreasing the expression levels of MMP-2 and MMP-9, which are required for cancer progression.

The EMT phenotype is tightly associated with cancer cell migration and invasiveness. Our results showed that down-regulation of EIF5B reversed the EMT phenotype. This suggested that EIF5B promoted HCC progression by inducing EMT. Previous documents have reported that activation of NF-κB signaling promoted EMT in HCC. Our data found that EIF5B regulated the NF-κB signaling pathway. We speculated that EIF5B induced EMT phenotype via the NF-κB signaling pathway. A subpopulation of cancer cells acquires stem cell-like properties during cancer progression and is termed as cancer stem cells (CSCs) [12]. Several studies have demonstrated that CSCs contribute to tumor relapse, metastasis, and chemotherapy drug resistance [18]. Our data demonstrated that down-regulation of EIF5B suppressed the CSC phenotype of HCC cells and increased their sensitivity to the cytotoxic drug, 5-FU.

The m6A modification regulates the stability of several mRNAs. In the present study, we analyzed whether IGF2BP3 contributed to the stability of EIF5B mRNAs. IGF2BP3 functions as a RNA binding protein and is involved in various cancer-related processes [19]. Our data demonstrated direct binding of IGF2BP3 to specific m6A sites in the EIF5B mRNA. The m6A modification regulated the half-life of EIF5B mRNAs. Taken together, our data suggested that EIF5B is a potential prognostic biomarker and therapeutic target in HCC.

Appendix

SUPPLEMENTARY TABLE 1 Differentially expressed genes(DEGs) between control and EIF5B-silenced HepG2 cells.

SUPPLEMENTARY TABLE 2 The demography of the HCC patients enrolled in the study.

## Data Availability

All the data generated for this study are available on request to the corresponding author.

## Abbreviations

HCC: hepatocellular carcinoma
EMT: epithelial-mesenchymal transition
CSC: cancer stem cell
5-FU: 5-fluorouracil
shRNA: short hairpin RNA
MTT: 3-(4,5-dimethylthiazol-2-yl)-2,5-diphenyl tetrazolium bromide
PBS: phosphate buffered saline
DEAB: Diethylaminobenzaldehyde
RIP: RNA immunoprecipitation
