# Supplementary material for: IGF2BP3-induced activation of EIF5B contributes to progression of hepatocellular carcinoma cells
Source: Oncol Res. 2023 Jan 5;30(2):77–87. doi: 10.32604/or.2022.026511 (PMC10207990; doi:10.32604/or.2022.026511)
Supplement: SUPPLEMENTARY TABLE 2 [file OncolRes-30-26511-s002.docx]

**Supplementary Table 2 The demography of the HCC patients enrolled in the study.**

| Patients | Gender | Age(years) |
| --- | --- | --- |
| 1 | Male | 78 |
| 2 | Female | 68 |
| 3 | Male | 59 |
| 4 | Male | 62 |
| 5 | Male | 66 |
| 6 | Female | 73 |
| 7 | Male | 76 |
| 8 | Male | 54 |
| 9 | Female | 57 |
| 10 | Female | 65 |
| 11 | Male | 63 |
| 12 | Male | 67 |
| 13 | Female | 79 |
| 14 | Male | 77 |
| 15 | Male | 77 |
| 16 | Male | 73 |
